# Lower‐Body Muscle Volumes Can Explain Half of the Variance in Sprint Speed Between Collegiate American Football Players

**DOI:** 10.1111/sms.70283

**Published:** 2026-04-18

**Authors:** Jack A. Martin, Mikel R. Joachim, Silvia S. Blemker, David A. Opar, Brett Mortensen, Bryan C. Heiderscheit, The HAMIR Study Group

**Affiliations:** ^1^ Department of Orthopedics and Rehabilitation, Badger Athletic Performance University of Wisconsin‐Madison Madison WI USA; ^2^ Springbok Analytics Charlottesville VA USA; ^3^ Department of Biomedical Engineering University of Virginia Charlottesville VA USA; ^4^ Sports Performance, Recovery, Injury and New Technologies (SPRINT) Research Centre, School of Behavioral and Health Sciences Australian Catholic University Melbourne VIC Australia; ^5^ BYU Athletics and Department of Exercise Sciences Brigham Young University Provo UT USA

**Keywords:** automated segmentation, inertial measurement units, machine learning, magnetic resonance imaging, running speed

## Abstract

Sprinting speed is critical for success across many sports. Lower‐body muscle size is important, but it is difficult to determine which muscles have the greatest influence on speed, and it is unclear how much variance in speed between athletes can be explained by variance in muscle size alone. The purpose of this study was to determine how much variance in on‐field sprinting speed can be explained by individual and combined lower‐body muscle volumes. Sprint speed was measured in sixty‐six collegiate football players using inertial measurement units, and muscle volumes were determined using magnetic resonance imaging. Coefficients of determination were calculated between speed and individual muscle volumes, summed volumes, and optimized linear combinations of volumes. Psoas major volume explained the most variance in sprint speed (50%), followed by gluteus medius (45%) and gluteus maximus (37%). The top sum of three muscle volumes (psoas major, gluteus medius, piriformis) explained an additional 7% of the variance in speed compared with psoas major alone. Similarly, the top optimized linear combination of muscle volumes (psoas major, gluteus medius, piriformis) explained 9% more variance than psoas major alone. These results indicate that psoas major volume is most predictive of sprinting speed, and that accounting for multiple muscle volumes may have limited additional benefit.

## 1 Introduction

Maximal sprinting speed is important across a wide variety of sports and is viewed as a primary predictor of success. Thus, it is of interest to understand which factors are most important in determining an individual's sprinting ability. Lower‐body muscle volumes are expected to play a large role in sprinting speed, and recent work has aimed to determine which muscles are associated with sprinting ability.

Sprinters have been found to have greater proximal muscle mass compared with non‐sprinters [1], which is associated with greater ability to generate the forces required to increase stride frequency and maintain short ground contact times in sprinting with little increase in leg moment of inertia and mechanical difficulty of swinging the leg compared with the increase in inertia from distal muscle mass [2]. Greater thigh muscle mass has primarily been associated with the biarticular muscles, such as rectus femoris, while monoarticular knee flexor/extensor mass appears to be similar between sprinters and non‐sprinters [3]. The proximal hip flexors and extensors, especially psoas major and gluteus maximus, have emerged as potentially the most important muscles in determining sprint speed [3, 4, 5, 6]. However, others have shown a relatively greater association between speed and hamstring size [7]. Though there are exceptions, prior work has generally focused on muscular contributions to sprinting speed in track athletes [1, 2, 3, 4, 5, 6, 7]. Yet speed is important across many sports, such that the determinants of sprint speed should be studied in additional populations. One of these sports is American football, where positional demands necessitate large differences in anthropometry and sprinting ability. Thus, it is possible that relationships between speed and muscularity may differ in this population, leading to results in contrast with previous studies. Studies in this area have been limited by the difficulty in feasibly quantifying many lower‐body muscle volumes across large numbers of individuals due to the need for manual muscle segmentation from magnetic resonance images. However, recent advances in automated segmentation via machine learning methods can enable rapid quantification of many muscle volumes in large numbers of subjects [8]. Implementation of these approaches makes it more feasible to assess relationships between athletic performance and muscle volumes throughout the entire lower body. Additionally, prior studies have only looked at variance explained by individual muscles' functional groups, or all muscles together, and have not accounted for the possibility that other subsets of muscles may actually be most important in determining sprint speed. Studying all muscles crossing the hip, knee, and ankle, both individually and in combination, can allow an estimate of a ceiling for how much variance in sprinting speed can be explained by lower‐body musculature.

The work presented here uses data collected as part of the HAMstring InjuRy (HAMIR) study [9] to assess the relationship between muscle volumes and sprinting ability. The purpose of this study was to determine how much variance in on‐field sprinting speed can be explained by variance in individual lower‐body muscle volumes and combinations of lower‐body muscle volumes in collegiate American football players.

## 2 Materials and Methods

The HAMIR study is a multisite prospective study investigating the etiology of hamstring strain injury in American football players [9]. The study includes both on‐field sprinting assessed with inertial measurement units (IMUs) and lower‐body muscle assessments performed using magnetic resonance imaging (MRI) and machine learning technology [8]. The study is approved by the Health Sciences Institutional Review Boards at the University of Wisconsin–Madison and each data collection site. The Collaborative Research Network of the American Medical Society for Sports Medicine serves as the Data Coordinating Center for the study.

### 2.1 Participants

Data from 66 male Division I collegiate football players participating in preseason baseline testing for the HAMIR study [9] were used for this analysis (21 offensive/defensive linemen: mass = 128.7 ± 15.9 kg [mean ± SD], height = 1.92 ± 0.06 m; 45 other position players: mass = 93.1 ± 9.6 kg, height = 1.85 ± 0.05 m). This analysis includes only participants from Brigham Young University (BYU) during the first year of data collection. All participants provided informed consent under a protocol approved by the BYU Institutional Review Board. To be eligible for participation in the study, players were required to be student‐athletes between the ages of 18 and 26 rostered on the varsity football team and have no history of malignant disease or contraindications to MRI. Players were additionally required to be cleared for full sport‐related activity at the time of testing.

### 2.2 On‐Field Sprinting and Speed Measurement

Sprinting speed measurement was performed on an indoor turf practice field after standard team warm‐up activities. Seven IMUs (Xsens MVN Awinda system, Movella, Enschede, Netherlands) were placed on the lower body of participants (sacrum, thighs, shanks, feet). The Xsens lower‐ body kinematic model was calibrated following the procedure outlined in the manufacturer's software (MVN 2021.2). During the free‐movement period, participants were instructed to perform a walking high‐knees task with emphasis on large sagittal plane range‐of‐motion in the hips, knees, and ankles. Kinematic model calibration success was confirmed by visual confirmation of correspondence between participant motion and motion of the avatar displayed in Xsens software.

Participants then performed two sprints (36.6 m for offensive and defensive line players, or 45.7 m for all other players). Sprint distances were chosen with input from team strength training staff, with the expectation that players in each group would reach their maximum speed within the prescribed distance while not sustaining additional unnecessary fatigue. IMU data were recorded at 100 Hz and processed within the Xsens software using the “HD reprocessing” option, and data were then exported for analysis in MATLAB. Position data from the pelvis segment of the Xsens kinematic model was used to determine sprint speed. First, the distance traveled from the starting point was calculated at each time step from the ground (transverse) plane pelvis position values. Distance traveled over the course of the sprint was then low‐pass filtered at 1 Hz using a fourth‐order Butterworth filter. This was then differentiated with respect to time to determine speed. Peak speed values were averaged across the two repeat trials, and the average value was used for subsequent analyses.

### 2.3 Muscle Volume Quantification

MRI examinations of the lumbo‐pelvic region and bilateral lower extremities were performed on participants prior to the start of pre‐season practices. MRI examinations were performed using a 3 T scanner (Magnetom Vida, Siemens, Malvern, PA, USA) and a flexible surface coil, and consisted of lumbo‐pelvic and bilateral lower extremity scans to quantify muscle volumes within the entire field of view. Lower extremity scans were axial multi‐slice 2D gradient echo fast SPGR sequences with parameters chosen to create high muscle signal and low fat signal (TR/TE: 839 ms/4.35 ms, FOV: 500 mm × 344 mm, 256 × 176 matrix, 2 × 2 × 5 mm voxel size, with fat suppression). Axial images were obtained contiguously from the 12th thoracic vertebra to below the ankle joint in acquisition sets of 40 images. The number of acquisition sets was determined on an individual basis based on players' body dimensions.

All visible lower extremity muscle volumes were determined by automated muscle segmentation using Springbok Analytics (Charlottesville, VA, USA) machine learning technology (Figure SS1) [8, 10]. The framework employed by Springbok was developed based on a large body of work [1, 11, 12], has previously been validated for quantifying muscle volumes in the lower‐body muscles included in this study [8, 10], and has been used across a variety of applications [13, 14, 15, 16, 17]. Springbok's algorithms are continually updated and refined based on training datasets generated using manual segmentation. The results of automated segmentations are visually vetted by trained segmenters for each scan, and muscle boundaries are manually corrected as necessary (Figure S2). Thus, the results of the automated segmentation process are expected to be substantially equivalent to those from manual segmentation. A repeatability analysis, including three segmenters vetting scans from four unique datasets not included in the training dataset, found dice similarity coefficients greater than 0.75 across all muscles and greater than 0.9 for the majority of muscles [10]. Volume differences between segmenters were less than 5 mL for all muscles in the lower body, indicating consistency in the vetting process. The vastus lateralis‐vastus intermedius boundary definition used by Springbok recognizes the presence of an internal aponeurosis within the vastus lateralis muscle based on dissection‐validated studies, [18, 19, 20] and this definition is applied consistently across scans for internal validity. Others have often treated this aponeurosis as the boundary between the two muscles [21, 22, 23], and this subject is currently under debate. Muscle volumes for each participant were normalized to the height‐body mass product [11] to account for the relationship between muscle volume and body size, and bilateral values were averaged for a total of 35 muscles.

### 2.4 Correlation Between Speed and Muscle Volumes

All correlation analyses were performed in MATLAB. First, the coefficient of determination (R^2^) was used to assess how much variance in sprint speed could be accounted for by variance in each normalized individual muscle volume. Next, R^2^ values were calculated between sprint speed and the summed volume of each possible combination of 3 muscles (6545 combinations from 35 muscles). Finally, a bounded optimization approach was used in MATLAB (‘fminsearchbnd’ [24]) to find linear coefficients, *x*, applied to each possible set of three individual muscle volumes (Equation 1), which maximized R^2^ values between summed volume, *V(x)*, and sprint speed, *S*, across all participants (Equation 2). Coefficients were bounded by −1 and 1, and initial guesses were 0.5. Bounds do not affect the final result, as only the ratios between coefficients are relevant here, and only served to narrow the range of values searched by the optimization function. After optimization, the coefficients were each divided by the magnitude of the largest, such that the largest coefficient would have a magnitude of 1 for each combination.

(1) $$V\left(x\right)=x_{1}V_{1}+x_{2}V_{2}+x_{3}V_{3}$$

(2) $$R^{2}\left(x\right)={\left(\frac{\sum_{i=1}^{n}\left[\left(V_{i}\left(x\right)-\bar{V}\left(x\right)\right)\left(S_{i}-\bar{S}\right)\right]}{\sqrt{\sum_{i=1}^{n}{\left(V_{i}\left(x\right)-\bar{V}\left(x\right)\right)}^{2}*\sum_{i=1}^{n}{\left(S_{i}-\bar{S}\right)}^{2}}}\right)}^{2}$$

## 3 Results

**FIGURE 1:**
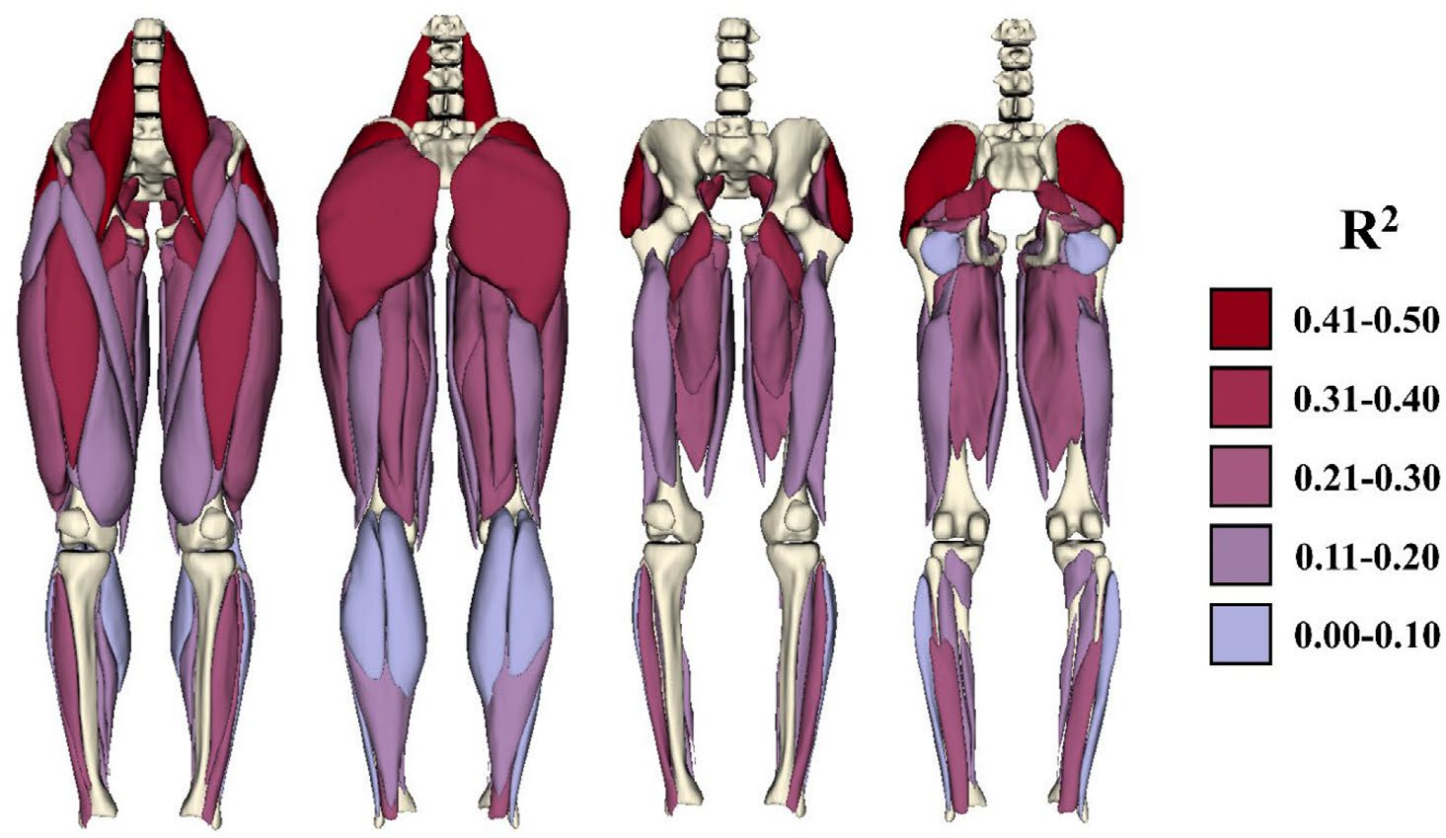
Variance in normalized volume of the individual muscles around the hip explained the most variance in peak sprinting speed. Superficial (left) and deep (right) muscles are colored based on the coefficient of determination (R^2^).

Players achieved sprinting speeds between 5.81 m·s^−1^ and 8.52 m·s^−1^ with a mean (± SD) of 7.41 ± 0.60 m·s^−1^ (offensive/defensive linemen: 6.89 ± 0.56 m·s^−1^; other position players: 7.63 ± 0.46 m·s^−1^; Figure S3). Peak speed occurred during 36.6 and 45.7 m sprints at mean (± SD) distances of 28.0 ± 3.9 m and 32.6 ± 6.8 m, respectively. Coefficients of determination between individual normalized muscle volumes and speed ranged from 0.04 to 0.50, with psoas major, gluteus medius, and gluteus maximus exhibiting the highest correlations (Figure 1, Table 1). All correlations were positive.

**TABLE 1:** Coefficient of determination (R^2^) between individual muscle volumes and peak sprinting speed, and normalized muscle volume (mean ± SD). Peroneus = combined peroneus brevis and longus; gemelli = combined gemelli superior and inferior; phalangeal extensors = combined extensor digitorum longus, extensor hallucis longus, and peroneus tertius.

Rank	Muscle	R^2^	Vol [cm^3^kg^−1^ m^−1^]	Rank	Muscle	R^2^	Vol [cm^3^kg^−1^ m^−1^]
1	Psoas Major	0.499	2.30 ± 0.43	19	Gemelli	0.202	0.12 ± 0.03
2	Gluteus Medius	0.451	2.43 ± 0.34	20	Popliteus	0.195	0.13 ± 0.03
3	Gluteus Maximus	0.365	8.44 ± 1.36	21	Vastus Medialis	0.190	4.27 ± 0.58
4	Pectineus	0.323	0.57 ± 0.09	22	Biceps Femoris Long Head	0.190	1.66 ± 0.33
5	Rectus Femoris	0.321	2.28 ± 0.43	23	Sartorius	0.188	1.41 ± 0.30
6	Piriformis	0.303	0.30 ± 0.09	24	Tensor Fasciae Latae	0.162	0.62 ± 0.15
7	Adductor Magnus	0.287	5.73 ± 0.88	25	Soleus	0.162	3.05 ± 0.43
8	Gluteus Minimus	0.280	0.68 ± 0.10	26	Tibialis Posterior	0.158	0.66 ± 0.15
9	Obturator Internus	0.276	0.15 ± 0.05	27	Flexor Digitorum Longus	0.156	0.17 ± 0.04
10	Semitendinosus	0.269	1.94 ± 0.38	28	Biceps Femoris Short Head	0.152	0.87 ± 0.17
11	Semimembranosus	0.266	2.02 ± 0.33	29	Gracilis	0.152	1.02 ± 0.19
12	Iliacus	0.239	1.37 ± 0.23	30	Obturator Externus	0.133	0.39 ± 0.07
13	Vastus Lateralis	0.232	7.95 ± 1.05	31	Vastus Intermedius	0.126	2.40 ± 0.36
14	Adductor Brevis	0.230	0.86 ± 0.13	32	Medial Gastrocnemius	0.090	1.85 ± 0.31
15	Flexor Hallucis Longus	0.225	0.50 ± 0.09	33	Lateral Gastrocnemius	0.077	1.08 ± 0.17
16	Phalangeal Extensors	0.219	0.60 ± 0.10	34	Peroneus	0.063	0.91 ± 0.15
17	Adductor Longus	0.214	1.45 ± 0.22	35	Quadratus Femoris	0.042	0.22 ± 0.06
18	Tibialis Anterior	0.205	0.85 ± 0.15				

The use of summed muscle volumes provided only modest gains in the ability to account for variance in sprinting speed. The combination of three muscle volumes explaining the greatest amount of variance was psoas major, gluteus medius, and piriformis (R^2^ = 0.57; Table 2). Each of the top 29 combinations included the psoas major, which was the individual muscle volume accounting for the most variance in speed. The first combination, not including psoas major, was gluteus medius, pectineus, and piriformis (R^2^ = 0.53).

**TABLE 2:** Variance in sprinting speed explained by simple sums of three muscle volumes. Sets of muscles displayed here include one of the top 10 individual muscle volumes (Table 1), and no muscle ranked higher, that is, the second row cannot include psoas major, and the third cannot include gluteus medius.

Rank	Muscle 1	Muscle 2	Muscle 3	R^2^
1	Psoas Major	Gluteus Medius	Piriformis	0.568
30	Gluteus Medius	Pectineus	Piriformis	0.530
662	Gluteus Maximus	Pectineus	Piriformis	0.425
317	Pectineus	Piriformis	Flexor Hallucis Longus	0.465
792	Rectus Femoris	Piriformis	Gluteus Minimus	0.414
591	Piriformis	Gluteus Minimus	Flexor Hallucis Longus	0.432
1134	Adductor Magnus	Semitendinosus	Soleus	0.367
843	Gluteus Minimus	Obturator Internus	Flexor Digitorum Longus	0.410
915	Obturator Internus	Popliteus	Flexor Hallucis Longus	0.404
1109	Semitendinosus	Semimembranosus	Iliacus	0.389

Optimized sums of volumes similarly provided only modest gains in the ability to account for variance in sprinting speed. The optimized combination of three muscle volumes explaining the greatest amount of variance was psoas major, gluteus medius, and piriformis (R^2^ = 0.59; Table 3). Each of the top 36 combinations included the psoas major. The first combination, not including psoas major, was gluteus medius, piriformis, and adductor magnus (R^2^ = 0.56). Muscle volumes were assigned negative coefficients in 6% of possible instances. The muscles most commonly assigned negative coefficients were the quadratus femoris (42% of appearances in a combination), the peroneus (29% of appearances), and the lateral gastrocnemius (26% of appearances). These are also the muscles with the lowest individual correlations with speed.

**TABLE 3:** Variance in sprinting speed explained by optimized linear combinations of three muscle volumes. Sets of muscles displayed here include one of the top 10 individual muscle volumes (Table 1), and no muscle ranked higher, that is, the second row cannot include psoas major, and the third cannot include gluteus medius.

Rank	Muscle 1	Coeff 1	Muscle 2	Coeff 2	Muscle 3	Coeff 3	R^2^
1	Psoas Major	0.35	Gluteus Medius	0.26	Piriformis	1.00	0.587
37	Gluteus Medius	0.42	Piriformis	1.00	Adductor Magnus	0.09	0.561
557	Gluteus Maximus	0.04	Obturator Internus	1.00	Popliteus	0.87	0.510
776	Pectineus	0.42	Obturator Internus	0.91	Popliteus	1.00	0.488
871	Rectus Femoris	0.14	Piriformis	1.00	Adductor Magnus	0.07	0.480
942	Piriformis	0.62	Adductor Magnus	0.06	Popliteus	1.00	0.475
901	Adductor Magnus	0.03	Obturator Internus	0.80	Popliteus	1.00	0.478
1 309	Gluteus Minimus	0.34	Obturator Internus	1.00	Sartorius	0.12	0.442
1 024	Obturator Internus	1.00	Iliacus	0.16	Phalengeal Extensors	0.32	0.466
2 136	Semitendinosus	0.11	Semimembranosus	0.14	Popliteus	1.00	0.395

**FIGURE 2:**
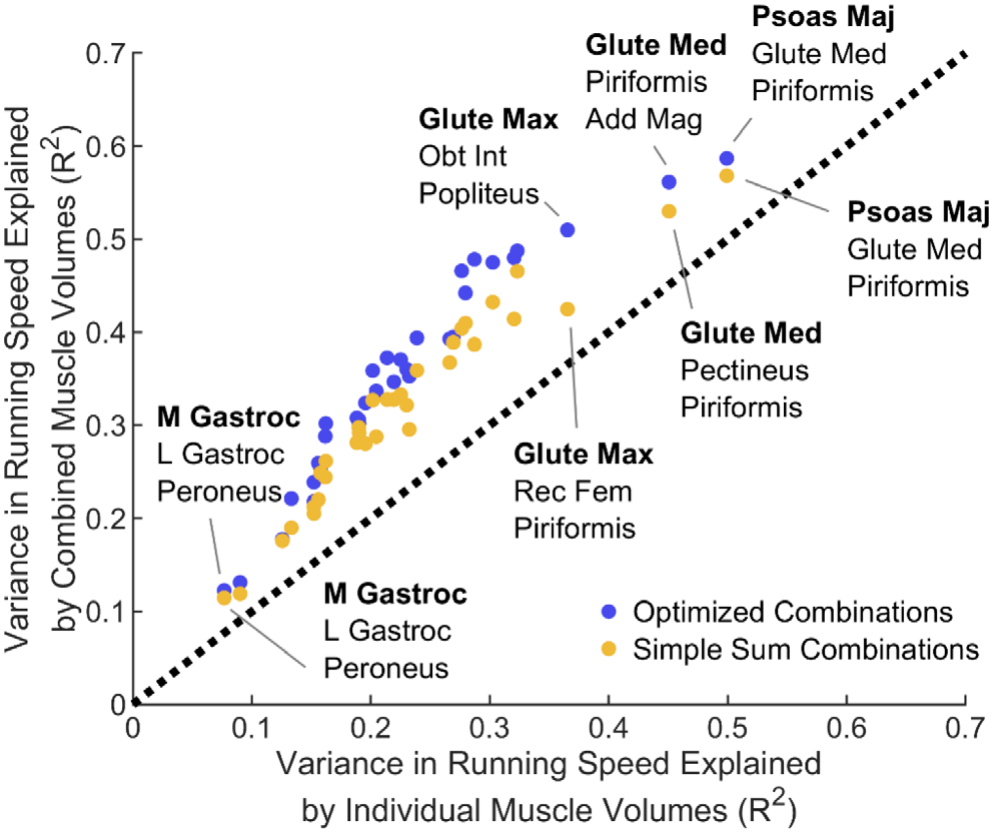
Variance in sprinting speed explained by simple sum or optimized combinations of muscle volumes (y‐axis) vs. variance explained by individual muscle volumes (x‐axis; Table 1). Displayed are the top combinations, including a given individual muscle and no muscles ranked higher, e.g., the point including gluteus medius (Glute Med) as the top muscle (bold) cannot include psoas major (Psoas Maj) because psoas major had a higher correlation with speed than did gluteus medius (Table 1). The dashed line indicates no improvement over the variance explained by individual muscle volumes. Neither combination approach explained much additional variance in sprinting speed compared with top performing individual muscles. Abbreviations: Add Mag, adductor magnus; Glute Max, gluteus maximus; L Gastroc, lateral gastrocnemius; M Gastroc, medial gastrocnemius; Obt Int, obturator internus; Rec Fem, rectus femoris.

Simple sum combinations of muscle volumes explained an additional 9% ± 3% of variance in speed compared with individual muscle volumes when considering the best performing combinations, including each muscle and no muscle with a higher individual correlation (Figure 2). Optimized linear combinations explained slightly more additional variance (12% ± 4%). Coefficients of determination for all combinations appear in Figure S4.

## 4 Discussion

The purpose of this study was to determine how much variance in maximal sprint speed can be explained by variance in individual normalized muscle volumes and combinations of normalized muscle volumes. Variance in the top individual muscle, psoas major, explained roughly half of the variance in speed, which is impressive given the number of factors expected to play a role in determining sprinting speed. However, combining multiple muscle volumes led to minimal improvement in the ability to account for differences in sprinting speed, with the top optimized combination that included psoas major only explaining 9% more variance in speed than psoas alone.

These results raise the question of why the psoas major is able to explain so much variance by itself. First, it is possible that the psoas is directly important for speed. Activity in the psoas has been shown to increase with speed, and the ability of the hip flexors to rapidly accelerate and swing the leg forward has been theorized to be an important factor for increasing speed [25]. Alternatively, it is possible that psoas size is related to total hip flexor and extensor development; both hip flexion and extension moments and powers are known to increase with speed as well [26, 27]. Finally, it is possible that relative psoas size is an indicator of a greater proximal‐distal mass ratio, where proximal mass increases strength while increasing leg moment of inertia to a lesser extent [1]. Here, proximal muscle volumes generally explained more variance than distal muscle volumes (Figure 1, Table 1). Additionally, distal muscles like the peroneus and gastrocnemii were often assigned negative coefficients in optimized linear combinations, meaning that a relatively smaller size of these muscles could be beneficial for speed. The latter two possibilities are both supported by relatively high correlations between psoas major volume and volume of other muscles acting on the hip (Figure S5).

The results of this study agree with prior work by Tottori et al., who found that mass‐normalized cross‐sectional areas of the psoas major and gluteus maximus were significantly larger in male sprinters than in non‐sprinters [6]. The results additionally agree with work by Ema et al., who found greater mass‐normalized hip flexor (psoas major and rectus femoris) volumes in male sprinters compared to untrained men [3], and Miller et al., who found that absolute and mass‐normalized hip extensor volumes were most different between male sprinters and non‐sprinters [5]. In the latter study, normalized gluteus maximus volume in particular explained 34% of the variance in sprinters' season‐best 100 m times. Conversely, the results presented here are not in agreement with those of Takahashi et al., who found that sprint velocity was associated with normalized semitendinosus volume, but not normalized gluteus maximus or adductor volumes in male sprinters [7]. The current findings also contrast with those of Xie et al., who found that normalized adductor longus and vastus medialis volumes were most associated with sprint performance in male basketball players [17]. However, Xie et al. examined speed achieved during 0–10 m and 10–20 m split distances, which may rely on different musculature compared with sprinting at maximal velocity.

Comparison between this study and prior work is interesting, given the different populations being studied. The American football players included in this study are subject to very different sport‐related demands compared with sprinters, who have been the focus of most work in this area. In particular, it is possible that contact‐related muscular demands may be associated with performance in early acceleration as well as greater strength in situations involving lower contraction velocities across many positional groups. Moreover, American football is associated with much greater muscle mass in the upper body than other sports, effectively decreasing mass‐normalized muscle volumes in the lower body, and linemen in particular are likely to have greater fat mass percentages. This is apparent when comparing the muscle volumes observed here with prior studies while correcting for height normalization. For example, the psoas major and iliopsoas complex volumes are similar or relatively smaller in this sample compared with sprinters and untrained men measured in prior studies [3, 5]. This observation extends to comparison of summed volumes of the vasti muscles as well, indicating that the effect is not isolated to muscles that are most important in sprint performance. Regardless, the general agreement of the results presented here with previous studies of sprinters suggests that muscular contributions to top speed may be independent of sport and observable across multiple sports despite sport‐specific demands.

Combined muscle volumes explained a maximum of 59% of the variance in sprinting speed across players in this study. This is consistent with results from a musculoskeletal modeling approach considering variations in muscle‐tendon properties, which predicts that leg muscle strength, and thus size, may be the most important factor in predicting sprint ability [28]. The amount of variance explained here exceeds the amount of variance in male 100 m sprint times explained by absolute and mass‐normalized gluteus maximus (44%, 34%), combined hip extensor (47%, 31%), and combined lower body (40%, 18%) muscle volumes [5]. This may be due to the ability of our approach to find more optimal muscle combinations for accounting for variance in sprint speed, or the use of linear combinations in addition to simple sums of muscle volumes. The greater variance explained may also be due to the difference in normalization approaches or a greater difficulty in accounting for differences in sprint times vs. peak sprint speeds. Alternatively, the 59% ceiling may be artificially high due to the number of possibilities that were considered. Regardless, our results indicate that at least 40% of the variance in sprint speed must be explained by other factors in this sample.

To further improve the ability to predict sprint speed, one must consider anthropometric and physiological factors. For instance, shorter Achilles tendon moment arms [29, 30], longer toes [30, 31], and longer forefoot bones [31] can improve sprinting ability by reducing the required muscle shortening velocity for a given ankle plantarflexion velocity. Additionally, greater muscle fascicle lengths can reduce shortening velocity requirements on the individual sarcomere level [30, 32, 33]. Muscle fiber‐type composition also plays a role, with a greater percentage of fast‐twitch fibers being advantageous for sprinting [34, 35].

There are several methodological choices that likely affected the results of this analysis. First, the combined muscle volume approach implemented in this study attempted to find the greatest portion of variance in sprinting speed explainable by variance in the combined volume of three muscles by trying every possible combination. A typical analysis would instead use an iterative approach [36], where a model is created using all muscle volumes, and individual muscles are successively removed from the model to find the best simple model. The latter approach reduces the risk of type I error and overestimation of explainable variance, while the former approach minimizes the risk of type II error and underestimation of explainable variance. The approach used here was appropriate for determining a ceiling on explainable variance. Thus, it is noteworthy that muscle combinations explained little additional variance in sprinting speed compared with individual muscles. The inability to explain much additional variance is likely due to relatively high intercorrelations between muscle volumes (Figure S5). Second, the number of muscles used in combination will affect the variance explained. We felt that three was an appropriate limit given the risk of overfitting with more predictors and the computational and interpretational challenges of using four or more muscles. For instance, there are 52 360 possible combinations of four muscles. Third, we chose to normalize muscle volumes to height and mass based on prior work showing that total lower‐body muscle mass is highly correlated with this product. This approach allows us to investigate the relationships between speed and relative muscle mass rather than absolute mass. This is essential given that investigations of absolute muscle mass will be confounded by body size. For example, the offensive linemen included in this study have the largest muscle masses and are also the slowest. Thus, an approach using absolute masses would tend to conclude that larger muscles are uniformly associated with lower running speeds, making the analysis uninteresting from a running biomechanics perspective. Finally, this study considered peak sprinting speed rather than sprint race times. We feel that studying speed directly is important, given that sprint times are additionally associated with maximal acceleration capacity.

The speeds measured in this study may have been affected by several factors. First, because IMU‐based speed measures are calculated from direct measures of acceleration, there is the potential for drift to affect results and add noise to the resulting relationships with muscle volume. However, the commercial Xsens software used for this study utilizes sensor fusion techniques to minimize this effect [37, 38]. Varying levels of effort and fatigue can also affect speed measures; however, individuals were provided with the same instructions and were taking part in similar training preseason programs prescribed by coaches. It is also important to consider the population that was included in this study. This study assessed collegiate American football players across a range of positions. While this allows for a large degree of variability in anthropometrics, all players are generally expected to possess a certain level of athletic ability, meaning that the results presented here may only be relevant to an athletic population. It is possible that the amount of explainable variance in speed or the relative importance of individual muscles would differ across position groups if these were assessed separately; however, the number of participants in this study was not sufficient for position‐specific analyses. Additionally, in contrast with prior studies, this study measured sprinting speeds on soft turf, which likely led to slower speeds compared with what would be observed on a track. Finally, it is important to note that this study and many others cited here have considered only male athletes, which highlights the general dearth of work examining performance in female athletes. It is possible that anthropometric differences between males and females may be associated with the different relative importance of muscle sizes to sprint speed. In this case, male athletes were studied because American college football was the focus of the HAMIR study. In the future, it is important that we and others incorporate data from female athletes as well.

## 5 Perspective

This analysis used automated muscle segmentation to circumvent the time demands of manual segmentation. These techniques are likely to become more widely used in the field, given the need for larger sample sizes, and careful vetting must be performed to ensure segmentation accuracy. The results of this analysis in American football players agree with prior studies in sprinters, showing that lower‐body muscle mass distribution is an important determinant of sprinting ability, and that the hip flexors and extensors may have the greatest influence on speed [3, 5, 6]. This agreement indicates that muscular determinants of speed may be consistent across sports despite differing sport‐specific demands. However, the amount of variance explainable by lower‐body musculature was capped at approximately 60%, and accounting for multiple muscles did little to exceed the variance explainable by key individual muscles contributing to hip flexion and extension. Thus, to predict sprinting ability, one must account for other factors such as muscle‐tendon geometry, muscle physiology, and anthropometrics. Still, the results of this analysis help us understand the relative importance of muscle mass and can inform training programs to improve speed. Further work should assess the relationship between muscle mass distribution and athletic ability in other populations, particularly female athletes.

## Author Contributions

J.A.M. Conceptualization, Formal analysis, Investigation, Methodology, Visualization, Writing – original draft, Writing – review and editing. MRS: Writing – review and editing. SSB: Funding acquisition, Writing – review and editing. BM: Funding acquisition, Writing – review and editing. DAO: Funding acquisition, Writing – review and editing. BCH: Funding acquisition, Supervision, Writing – review and editing.

## Funding

This work was supported by the National Football League. This study was funded by the National Football League. The NFL had no role in the design of this study and has not had any role during its execution, analyses, interpretation of the data, or our decision to submit results.

## Ethics Statement

The study is approved by the Health Sciences Institutional Review Boards at the University of Wisconsin–Madison (IRB 2021–1420) and each data collection site.

## Consent

All participants provided informed consent under a protocol approved by the BYU Institutional Review Board.

## Conflicts of Interest

Drs. Blemker (Chief Science Officer) and Heiderscheit (advisory board member) declare potential competing interests related to this work due to their respective roles with Springbok Analytics. The other authors declare no competing interests.

## Supporting information


**Figure S1:** Representative magnetic resonance imaging slices with Springbok segmentations. Abbreviations: PSO, psoas major; IL, iliacus; GMIN, gluteus minimus; GMED, gluteus medius; GMAX, gluteus maximus; PEC, pectineus; SA, sartorius; TFL, tensor fasciae latae; QF, quadratus femoris; OBT, obturator internus; GR, gracilis; AB, adductor brevis; AL, adductor longus; RF, rectus femoris; VI, vastus intermedius; VL, vastus lateralis; VM, vastus medialis; PEC, pectineus; ST, semitendinosus; AM, adductor magnus; BFLH, biceps femoris long head; SM, semimembranosus; BFSH, biceps femoris short head; TA, tibialis anterior; PE, phalangeal extensors; PR, peroneus; SOL, soleus; GMED, medial gastrocnemius; TP, tibialis posterior; FDL, flexor digitorum longus.
**Figure S2:** Example segmentation correction (dashed circle) during vetting. In this case, an artifact on the left side of the image interfered with the automated segmentation. The segmenter vetting the scan was required to make a correction here, adding the darker green area. Abbreviations: VL, vastus lateralis.
**Figure S3:** Distributions of peak speed across participants (0.5 m/s bins, mean ± SD) show that linemen ran substantially slower than other position players on average.
**Figure S4:** Variance in sprinting speed explained by simple sum or optimized combinations of muscle volumes (y‐axis) vs. variance explained by individual muscle volumes (x‐axis; Table 1). Displayed are all combinations including a given individual muscle and no muscles ranked higher, e.g., the points including gluteus medius (group appearing second from right) as the top muscle cannot include psoas major. The dashed line indicates no improvement over the variance explained by individual muscle volumes. Data points are plotted with random jitter up to ±0.01 in each direction to improve visualization.
**Figure S5:** Coefficients of determination (R‐Squared) between normalized individual muscle volumes. Muscles are ordered from highest to lowest correlation with psoas major (left‐to‐right). Abbreviations: Psoas Maj, psoas major; Glute Med, gluteus medius; Glute Max, gluteus maximus; Add Long, adductor longus; Semiten, semitendinosus; Add Brev, adductor brevis; Add Mag, adductor magnus; Rectus Fem, rectus femoris; Obturator Ext, obturator externus; Semimem, semimembranosus; Biceps Fem LH, biceps femoris long head; Glute Min, gluteus minimus; Vastus Lat, vastus lateralis; Biceps Fem SH, biceps femoris short head; Phal Ext, phalangeal extensors; Tib Ant, tibialis anterior; FHL, flexor hallucis longus; Obturator Int, obturator internus; Gastroc Med, medial gastrocnemius; Tib Post, tibialis posterior; Vastus Med, vastus medialis; Gastroc Lat, lateral gastrocnemius; Quadratus Fem, quadratus femoris; Vastus Int, vastus intermedius; FDL, flexor digitorum longus; TFL, tensor fasciae latae.

## Data Availability

The data that support the findings of this study are available from the corresponding author upon reasonable request. Data requestors will be asked to sign a data use agreement with the data coordinating center for the study: American Medical Society for Sports Medicine.
