# Supplementary material for: Lower‐Body Muscle Volumes Can Explain Half of the Variance in Sprint Speed Between Collegiate American Football Players
Source: Scand J Med Sci Sports. 2026 Apr 18;36:e70283. doi: 10.1111/sms.70283 (PMC13091570; doi:10.1111/sms.70283)
Supplement: Supplementary file 1 — Figure S1: Representative magnetic resonance imaging slices with Springbok segmentations. Abbreviations: PSO, psoas major; IL, iliacus; GMIN, gluteus minimus; GMED, gluteus medius; GMAX, gluteus maximus; PEC, pectineus; SA, sartorius; TFL, tensor fasciae latae; QF, quadratus femoris; OBT, obturator internus; GR, gracilis; AB, adductor brevis; AL, adductor longus; RF, rectus femoris; VI, vastus intermedius; VL, vastus lateralis; VM, vastus medialis; PEC, pectineus; ST, semitendinosus; AM, adductor magnus; BFLH, biceps femoris long head; SM, semimembranosus; BFSH, biceps femoris short head; TA, tibialis anterior; PE, phalangeal extensors; PR, peroneus; SOL, soleus; GMED, medial gastrocnemius; TP, tibialis posterior; FDL, flexor digitorum longus. Figure S2: Example segmentation correction (dashed circle) during vetting. In this case, an artifact on the left side of the image interfered with the automated segmentation. The segmenter vetting the scan was required to make a correction here, adding the darker green area. Abbreviations: VL, vastus lateralis. Figure S3: Distributions of peak speed across participants (0.5 m/s bins, mean ± SD) show that linemen ran substantially slower than other position players on average. Figure S4: Variance in sprinting speed explained by simple sum or optimized combinations of muscle volumes (y‐axis) vs. variance explained by individual muscle volumes (x‐axis; Table 1). Displayed are all combinations including a given individual muscle and no muscles ranked higher, e.g., the points including gluteus medius (group appearing second from right) as the top muscle cannot include psoas major. The dashed line indicates no improvement over the variance explained by individual muscle volumes. Data points are plotted with random jitter up to ±0.01 in each direction to improve visualization. Figure S5: Coefficients of determination (R‐Squared) between normalized individual muscle volumes. Muscles are ordered from highest to lowest correlation with [file SMS-36-e70283-s001.docx]

**Lower Limb Muscle Volumes Can Explain Half of the Variance in Sprint Speed between Collegiate Football Players: Supplementary Material**

Jack A. Martin

Department of Orthopedics and Rehabilitation, Badger Athletic Performance Program, Department of Mechanical Engineering

University of Wisconsin-Madison

6136 Medical Foundation Centennial Building; 1685 Highland Ave; Madison, WI 53705

Email: jamartin8@wisc.edu

Mikel R. Joachim

Department of Orthopedics and Rehabilitation, Badger Athletic Performance Program

University of Wisconsin-Madison

Silvia S. Blemker

Springbok Analytics

Department of Biomedical Engineering, Department of Mechanical Engineering

University of Virginia

David A. Opar

Sports Performance, Recovery, Injury and New Technologies (SPRINT) Research Centre

School of Behavioral and Health Sciences

Australian Catholic University

Brett Mortensen

BYU Athletics

Department of Exercise Sciences

Brigham Young University

Bryan C. Heiderscheit

Department of Orthopedics and Rehabilitation, Badger Athletic Performance Program, Department of Biomedical Engineering

University of Wisconsin-Madison

The HAMIR Study Group

Geoffrey Baer, Craig Buckley, Kyle Costigan, Shauna Drew, Duffy Eberhardt, Kurrel Fabian, Herman Feller, Erin Hammer, Danielle Heidt, Kenneth Lee, Brian Lund, Michael Moll, Jennifer Sanfilippo, Shaun Snee, Claire Tanaka, Ty Taylor, John Wilson, Devin Woodhouse, Yi-Chung Lin, Jack Hickey, Nirav Maniar, Ashleigh Homer, Ryan Timmins, Matthew Cousins, Olivia DuCharme, Xue Feng, Scott Magargee, Craig Meyer, Anthony Nguyen, Lara Riem, Robin West, Steven Allen, Dain Allred, Anthony Beutler, Dustin Bruening, Darren Campbell, A. Wayne Johnson, Camille Nguyen, Emma Remington, Annie A. Smedley, Joshua K. Sponbeck, Stephanie Kliethermes & Sarah Sund


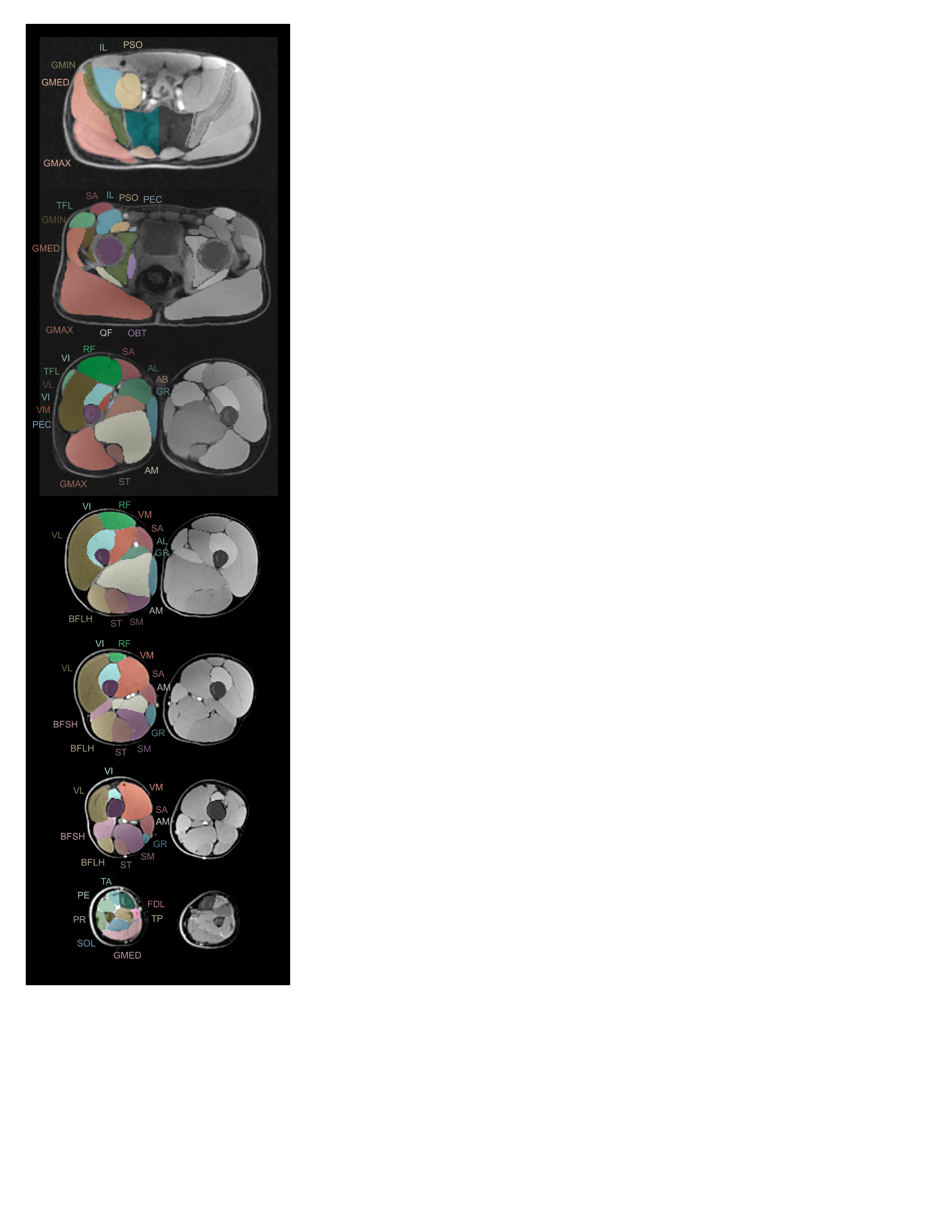


**Supplementary Figure 1.** Representative magnetic resonance imaging slices with Springbok segmentations. Abbreviations: PSO, psoas major; IL, iliacus; GMIN, gluteus minimus; GMED, gluteus medius; GMAX, gluteus maximus; PEC, pectineus; SA, sartorius; TFL, tensor fasciae latae; QF, quadratus femoris; OBT, obturator internus; GR, gracilis; AB, adductor brevis; AL, adductor longus; RF, rectus femoris; VI, vastus intermedius; VL, vastus lateralis; VM, vastus medialis; PEC, pectineus; ST, semitendinosus; AM, adductor magnus; BFLH, biceps femoris long head; SM, semimembranosus; BFSH, biceps femoris short head; TA, tibialis anterior; PE, phalangeal extensors; PR, peroneus; SOL, soleus; GMED, medial gastrocnemius; TP, tibialis posterior; FDL, flexor digitorum longus.


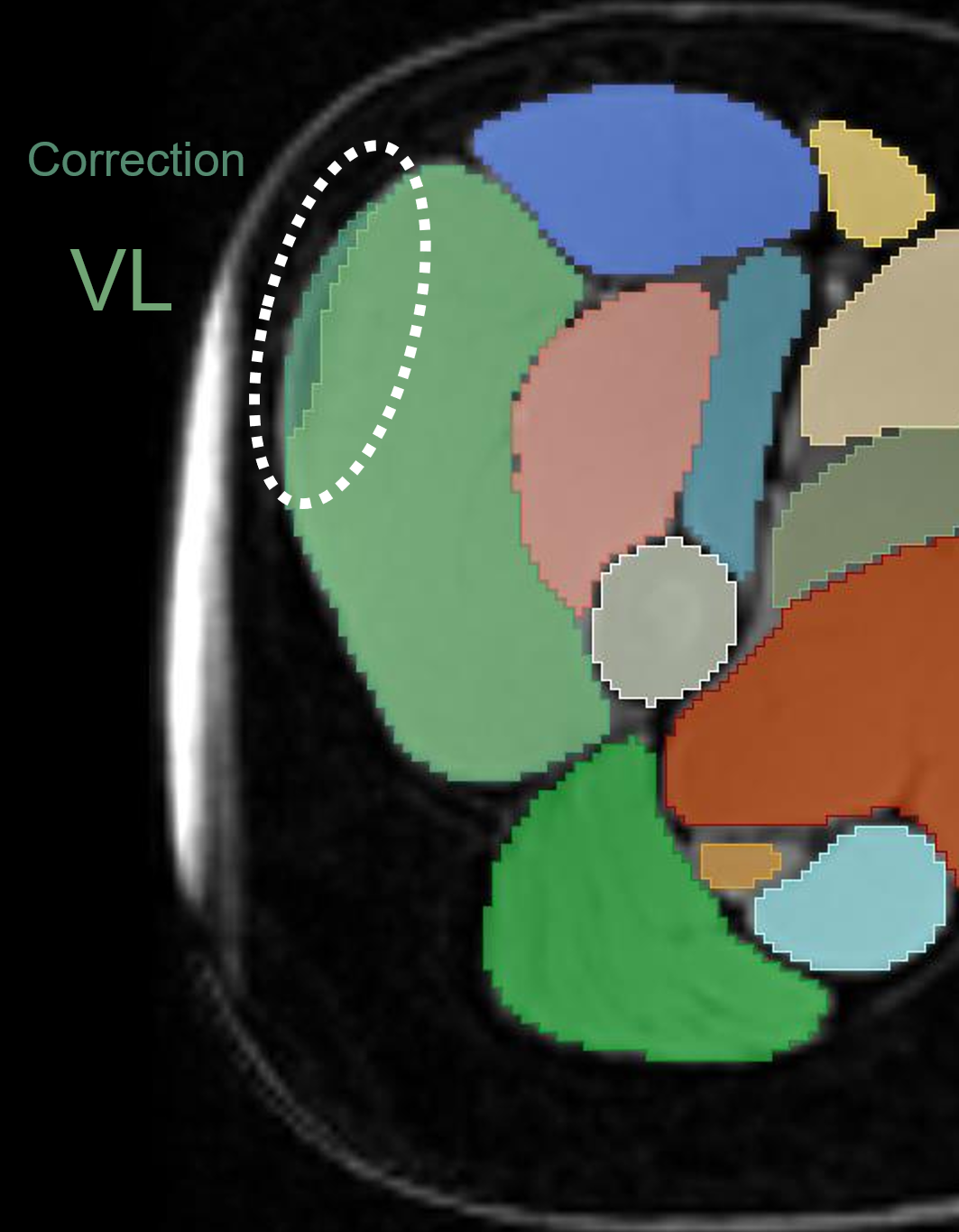


**Supplementary Figure 2.** Example segmentation correction (dashed circle) during vetting. In this case, an artifact on the left side of the image interfered with the automated segmentation. The segmenter vetting the scan was required to make a correction here, adding the darker green area. Abbreviations: VL, vastus lateralis.


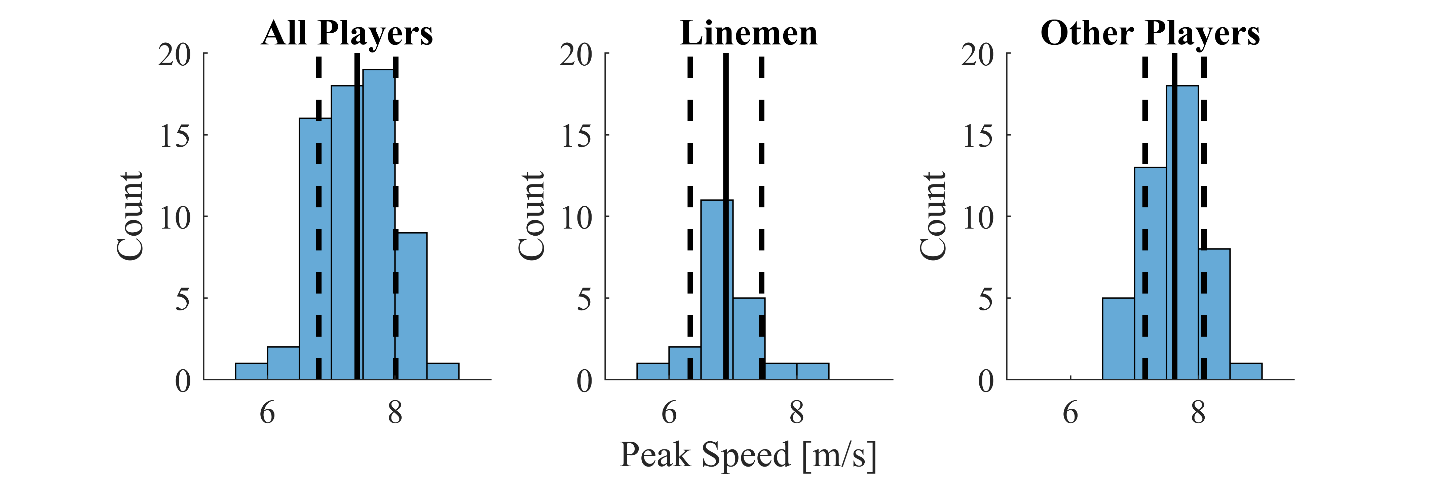


**Supplementary Figure 3.** Distributions of peak speed across participants (0.5 m/s bins, mean ± SD) show that linemen ran substantially slower than other position players on average.


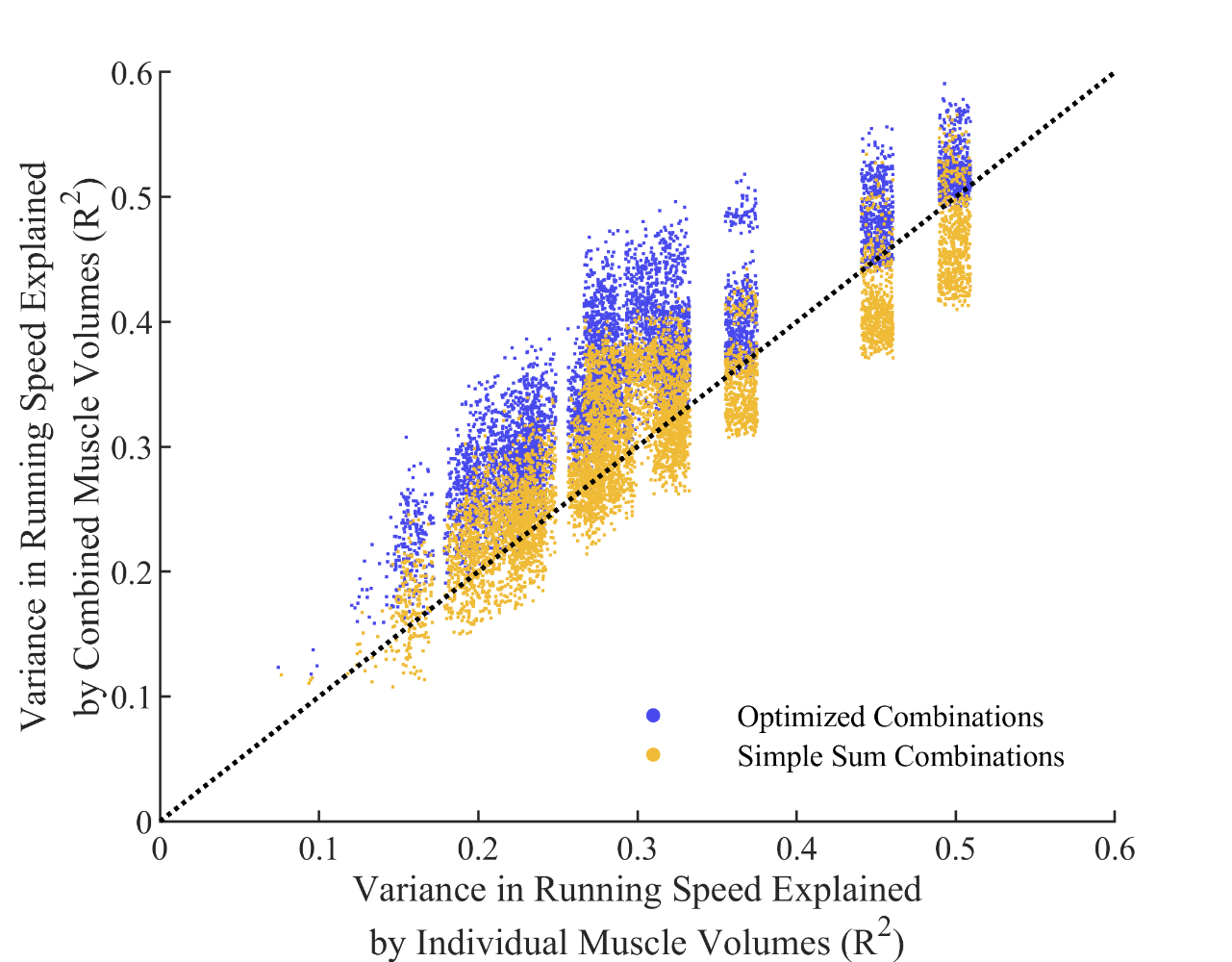


**Supplementary Figure 4.** Variance in sprinting speed explained by simple sum or optimized combinations of muscle volumes (y-axis) vs. variance explained by individual muscle volumes (x-axis; Table 1). Displayed are all combinations including a given individual muscle and no muscles ranked higher, e.g., the points including gluteus medius (group appearing second from right) as the top muscle cannot include psoas major. Dashed line indicates no improvement over variance explained by individual muscle volumes. Data points are plotted with random jitter up to ± 0.01 in each direction to improve visualization.


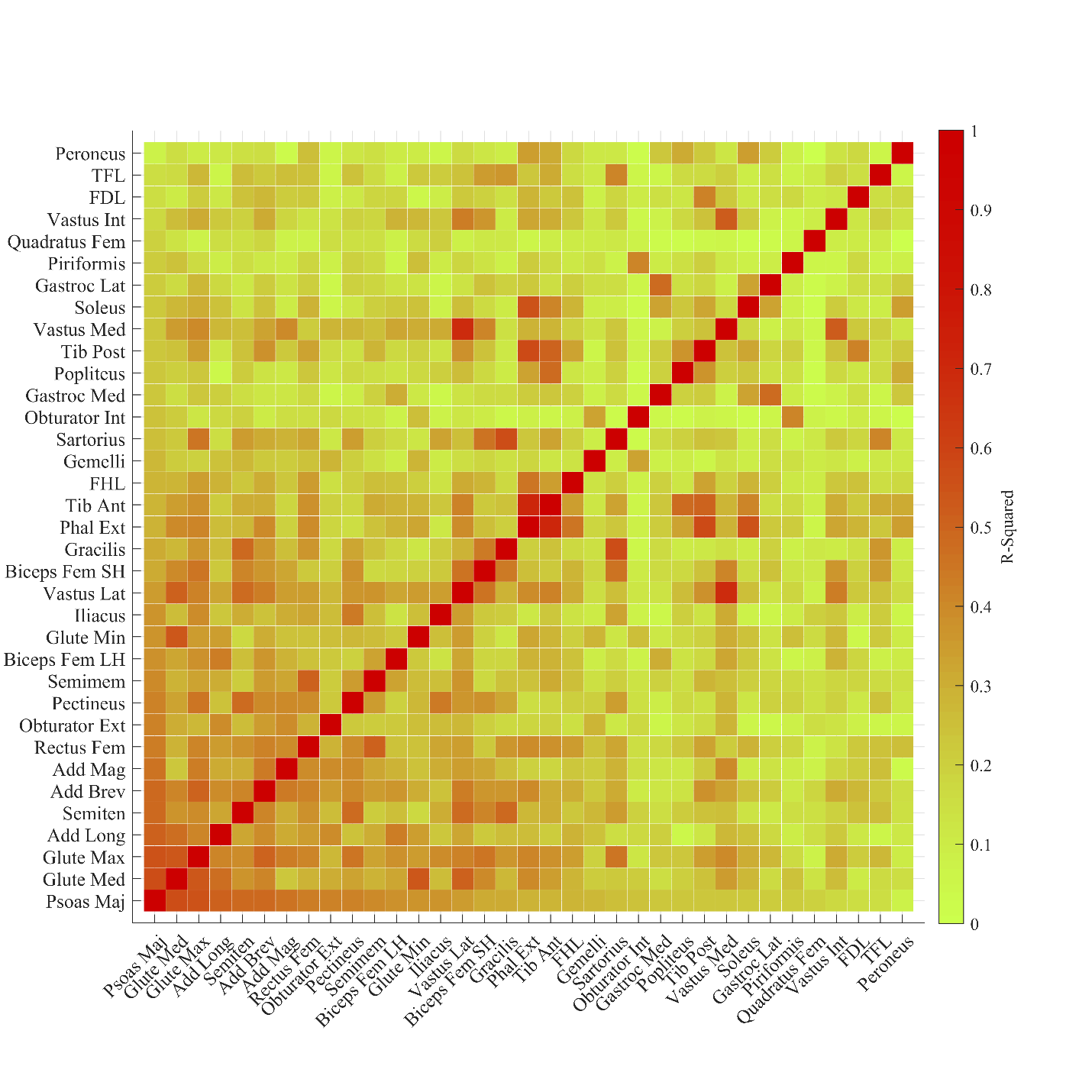


**Supplementary Figure 5.** Coefficients of determination (R-Squared) between normalized individual muscle volumes. Muscles are ordered from highest to lowest correlation with psoas major (left-to-right). Abbreviations: Psoas Maj, psoas major; Glute Med, gluteus medius; Glute Max, gluteus maximus; Add Long, adductor longus; Semiten, semitendinosus; Add Brev, adductor brevis; Add Mag, adductor magnus; Rectus Fem, rectus femoris; Obturator Ext, obturator externus; Semimem, semimembranosus; Biceps Fem LH, biceps femoris long head; Glute Min, gluteus minimus; Vastus Lat, vastus lateralis; Biceps Fem SH, biceps femoris short head; Phal Ext, phalangeal extensors; Tib Ant, tibialis anterior; FHL, flexor hallucis longus; Obturator Int, obturator internus; Gastroc Med, medial gastrocnemius; Tib Post, tibialis posterior; Vastus Med, vastus medialis; Gastroc Lat, lateral gastrocnemius; Quadratus Fem, quadratus femoris; Vastus Int, vastus intermedius; FDL, flexor digitorum longus; TFL, tensor fasciae latae.
